# Direct Characterization of Transcription Elongation by RNA Polymerase I

**DOI:** 10.1371/journal.pone.0159527

**Published:** 2016-07-25

**Authors:** Suleyman Ucuncuoglu, Krysta L. Engel, Prashant K. Purohit, David D. Dunlap, David A. Schneider, Laura Finzi

**Affiliations:** 1 Physics Department, Emory University, Atlanta, GA, 30322, United States of America; 2 Biochemistry and Molecular Genetics, University of Alabama at Birmingham, Birmingham, AL, 35294, United States of America; 3 Department of Mechanical Engineering and Applied Mechanics, University of Pennsylvania, Philadelphia, PA, 19104, United States of America; Institute of Molecular Genetics IMG-CNR, ITALY

## Abstract

RNA polymerase I (Pol I) transcribes ribosomal DNA and is responsible for more than 60% of transcription in a growing cell. Despite this fundamental role that directly impacts cell growth and proliferation, the kinetics of transcription by Pol I are poorly understood. This study provides direct characterization of *S*. *Cerevisiae* Pol I transcription elongation using tethered particle microscopy (TPM). Pol I was shown to elongate at an average rate of approximately 20 nt/s. However, the maximum speed observed was, in average, about 60 nt/s, comparable to the rate calculated based on the *in vivo* number of active genes, the cell division rate and the number of engaged polymerases observed in EM images. Addition of RNA endonucleases to the TPM elongation assays enhanced processivity. Together, these data suggest that additional transcription factors contribute to efficient and processive transcription elongation by RNA polymerase I *in vivo*.

## Introduction

In all living cells there is at least one multi-subunit RNA polymerase. In prokaryotes, a single RNA polymerase synthesizes all cellular RNA. In contrast, eukaryotes utilize at least three nuclear RNA polymerases. Each one has a unique set of factors required for the initiation of transcription and target genes [1]. RNA polymerase I (Pol I) transcribes ribosomal DNA; RNA polymerase II (Pol II) synthesizes all mRNA and some noncoding RNA; whereas RNA polymerase III (Pol III) produces tRNAs and the 5S rRNA.

Since the discovery of specialized RNA polymerases in eukaryotes [2], many labs have described key features and activities of these enzymes. However, due to its diverse set of target genes and clear connection to development, the majority of studies in eukaryotic transcription have focused on RNA polymerase II. Thousands of elegant studies employing a wide array of approaches have described Pol II transcription and its regulation. These studies have uncovered many trans-acting factors that contribute to efficiency and regulation of transcription elongation by Pol II [3]. Furthermore, detailed biochemical studies using ensemble assays and single-molecule strategies have yielded many insights into the elongation kinetics of Pol II transcription (e.g. [4, 5]). Comparatively little is known about the details of transcription by Pol I, although multi-subunit RNA polymerases are well conserved and Pol II has been used as a model for the other eukaryotic RNA polymerases.

Pol I is uniquely responsible for synthesis of the three largest rRNA species. Multiple studies over many years have demonstrated that ribosome synthesis is energetically costly for cells and directly connected to the rates of cell growth and proliferation [6]. Despite the intrinsic importance of Pol I transcription, the details of its activity remain poorly understood. In addition, several recent findings suggest that transcription elongation by Pol I is distinctly different from that of Pol II. In 2007, the Nomura lab showed that selective impairment of Pol I transcription elongation results in rampant defects in pre-rRNA processing and ribosome biogenesis [7]. Thus, transcription elongation by Pol I is functionally coupled to pre-rRNA processing. Furthermore, a more recent study from the Schneider and Kaplan laboratories discovered opposite phenotypes of identical mutations in highly conserved amino acid residues of Pols I and II [8]. The authors concluded that these enzymes have evolved unique elongation properties, perhaps to better suit each enzyme’s unique cellular roles. In addition, the Cramer and Mueller labs recently solved high resolution crystal structures of yeast Pol I and found that the enzyme is dramatically different from Pol II in a number of critical domains [9, 10]. All of these data suggest that eukaryotic RNA polymerases have evolved unique enzymatic properties, and in order to fully understand eukaryotic gene expression, the unique transcription elongation properties of Pol I must be analysed more carefully.

Pol I has also drawn interest as a potential target for anti-cancer chemotherapy [11, 12]. Recent work from a number of labs suggests that developing a clear understanding of Pol I and its regulation may hold considerable clinical value. Based on the knowledge that ribosome synthesis rates are proportional to growth rate, a number of groups have identified molecules that directly inhibit Pol I transcription. Both *in vitro* and in pre-clinical animal models, inhibition of Pol I transcription resulted in selective inhibition of tumor cell growth [11, 13]. Thus, the therapeutic potential of Pol I inhibition is high and a deeper understanding of Pol I transcription is warranted.

In this study we have developed a single molecule assay for transcription elongation by RNA polymerase I. The assay employs promoter- and factor-dependent transcription initiation from the native promoter for Pol I. We assembled a polystyrene microsphere tethered by a DNA template attached to nucleotide triphosphate deprived transcription elongation complexes adsorbed on a glass slide. Elongation resumed upon addition of the missing nucleotide triphosphate and altered the length of the tether, which could be monitored using tethered particle motion (TPM) analysis. In order to overcome challenges presented by low numbers of active, adsorbed elongation complexes, we multiplexed our tethered particle instrumentation to track dozens of complexes simultaneously. In these conditions, a small fraction of Pol I elongation complexes traversed the entire DNA template. This was also observed in ensemble, biochemical assays, thus, Pol I is not highly processive *in vitro*, despite its exceptional processivity in living cells. Nonetheless, the maximum enzyme velocity measured using TPM approached the values estimated for *in vivo* conditions based on electron microscopy studies [14]. Furthermore, RNases increased Pol I processivity, particularly in the region from the halt site (position 556) to position 1000 but also up to position 1400 of the 2500 bp-long DNA construct. These data suggest that one or more additional factors enhance processivity of Pol I *in vivo*, perhaps by minimizing the formation of RNA/DNA hybrids in the upstream template.

## Materials and Methods

### Transcription reagent preparation

**Fig 1:**
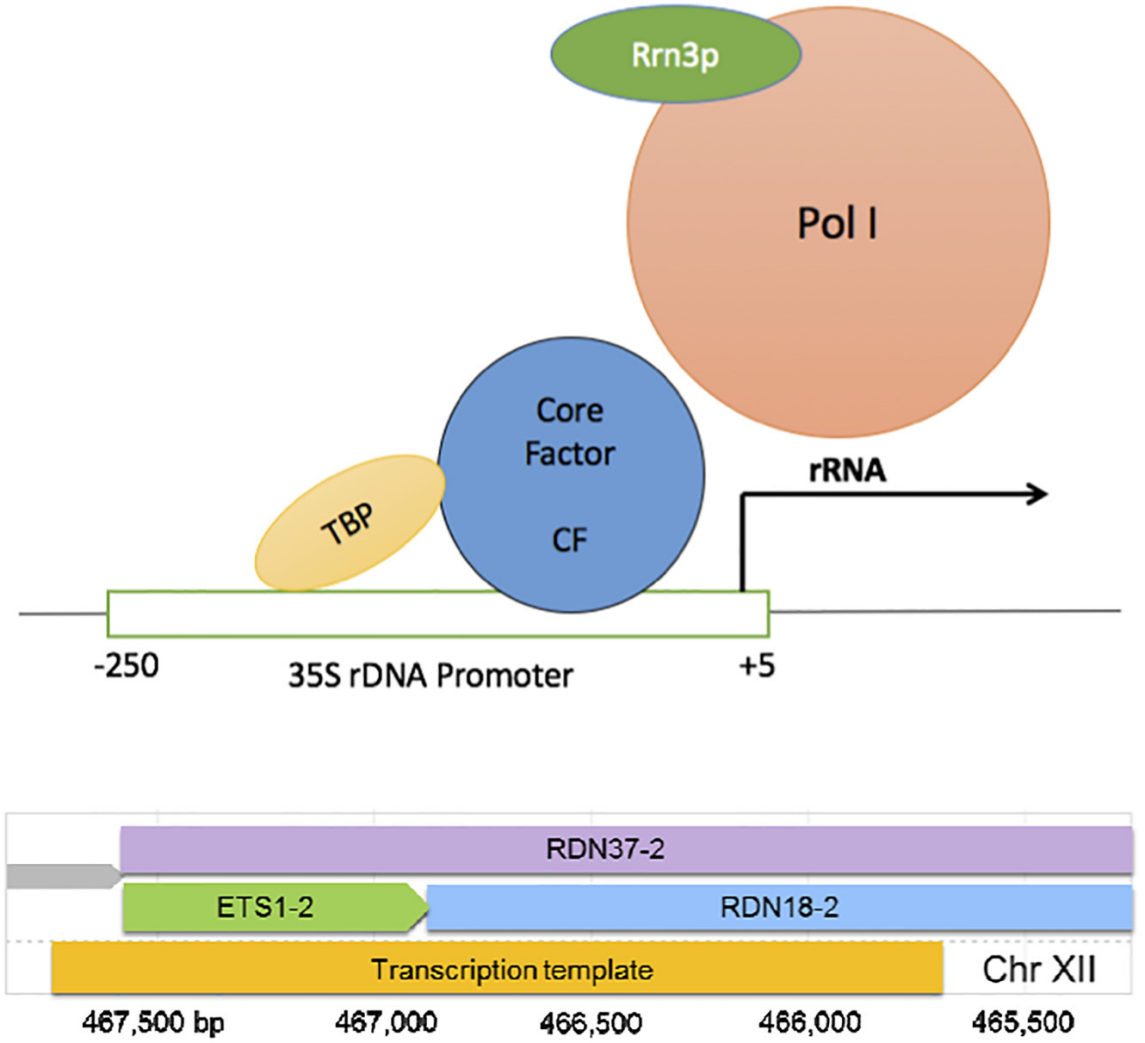
DNA and protein components. A diagram (not to scale) of the rDNA elements and proteins used in the transcription assays reported here (top). A diagram of the chromosomal context of the rDNA fragment used in the TPM measurements of RNA Pol I elongation (bottom). Note that since Pol I only transcribes rDNA, the DNA tether selected for our experiments was a portion of the natural Pol I template.

All DNA templates included *Saccharomyces cerevisiae* rDNA sequences (referred to as “yeast” herein) and were amplified from the plasmid template pNOY745 by polymerase chain reaction (PCR) using high fidelity DNA polymerase (Phusion, New England BioLabs Inc., Ipswich, MA) and the following unlabeled and 5’-digoxigenin-labeled primers (Integrated DNA Technologies Inc., Coralville, Iowa): 5’-tcactccaccaactgagaaacg and 5’(dig)-atggtgaaagttccctcaagaat. PCR products were purified using a kit, (Qiagen; Germantown, MD). Schematic representations of the DNA constructs used are shown in S1 Fig. The upper panel of Fig 1 shows the main rDNA elements and protein factors used in the elongation experiments described here. Core factor, Rrn3, TBP and RNA polymerase I were purified as described previously [15]. Briefly, Core factor, Rrn3, and TBP were overproduced in *Escherichia coli* and purified. Pol I was purified from *Saccharomyces cerevisiae*, taking advantage of a dual epitope tag (his-HA) on the C-terminus of the A135 subunit. The lower panel of Fig 1 shows the chromosomal context (yeast) of the rDNA fragment used.

### TPM Microchamber preparation

Microchambers were prepared as detailed in Kumar *et al* [16]. In brief, a 22 X 22 mm, no. 1 (Fisherbrand, cat. # 12-548-B, Thermo Fisher Scientific, Waltham, MA) and a 50 x 24 mm coverslip (Fisherbrand, cat. # 12-545-F) were cleaned with laboratory detergent, rinsed with tap water and then de-ionized water before storage under ethanol. Double-sided tape 2 mm wide and 22 mm long was used to glue the two coverslips and form a channel lined with vacuum grease. (S2 Fig).

Chambers were rinsed with two or more volumes of 20 mM Tris-acetate pH 7.9, 2 mM DTT, 100 mM potassium glutamate, 8 mM magnesium acetate and 0.5 mg/ml α-casein (TR buffer) by depositing a drop of buffer at one opening, allowing the liquid to enter by capillary action, and using a laboratory tissue to wick solution from the opposite opening and draw solution into/through the microchamber. 40 μl of 1:1000 solution of anti-HA antibody (12CA5; stock concentration of 1 mg/ml, Abcam, Cambridge, MA) diluted in TR buffer was added to the chamber and incubated at least 2 hours at room temperature or overnight at 4 degrees. Before use, the chambers were washed with 400 μl of TR buffer and incubated for 5 minutes.

### Sample Preparation

To form pre-initiation complexes, 0.5 pmol of DNA template, 0.3 pmol of core factor (CF), and 0.3 pmol of TATA binding protein (TBP) were added to 43.1 μl of TR buffer supplemented with 0.5 mg/ml α-Casein. The solution was incubated for 3 minutes at room temperature to permit assembly of the pre-initiation complex on the DNA. Then, 0.25 pmol of Pol I-Rrn3p complex was added and incubated for 10 minutes. Pol I carried a triple hemagglutinin (3-HA) epitope on the C-terminus of the A135 subunit. One μl of a 10 mM ATP, UTP, and GTP mixture was added to give a final volume of 50 μl which was incubated for 3 minutes to initiate transcription and produce synchronized elongation complexes halted at position 56, at the first guanine in the template. Twenty μl of solution containing halted complexes were introduced in the microchamber and incubated for 15 minutes to facilitate the binding of 3-HA tagged Pol I to the anti-HA-coated chamber surface. The microchamber was then gently flushed with 400 μl of TR buffer to remove the unbound complexes. After 5 minutes, 25 μl of 240 nm anti-dig coated beads (Indicia Diagnostics, Oullins, France), previously diluted 50X in TR buffer and vortexed for 15 minutes, were added. Finally, excess beads were gently flushed from the chamber with 400 μl of TR buffer. In some experiments, 0.5 μl (2.5u) of RNase H (New England BioLabs Inc., Ipswich, MA, M0297S) or 2.5u of RNase T1 and 1 μg of RNase A/T1 (Thermo Fisher Scientific, Grand Island, NY, EN0551) were introduced along with NTPs. Sixty five different Pol I-DNA complexes in 27 separate microchambers exhibited elongation events in the absence of RNase. Fourteen (13) more Pol I-DNA complexes in 11 (8) separate microchambers exhibited elongation events in the presence of RNase H (RNase A/T1).

### Optical Setup

Differential interference contrast (DIC) microscopy was used to monitor the Brownian motion of tethered beads projected in the image plane. The DIC image (S3 Fig) was magnified 40X (63X objective, and 0.63X camera mount (Leica, Wetzlar, Germany)).

In order to increase the data output per experiment (multiplexing), we decreased the magnification of our microscope system compared to previous studies [17, 18]. The larger field of view allowed visualization of up to 100 particles in each field, but required a larger pixel format camera with data transfer fast enough for video steaming to disk storage and off-line, multiplexed particle tracking (see Results).

### Instrumentation

A Gigabit Ethernet (gigE) camera (CM-140GE, JAI, Copenhagen, Denmark) was used to acquire images with 1390 X 1040 resolution at 30 frames per second. Streaming videos at high-resolution demanded data rates up to 125 MB/s, and category 6 cables were utilized between the camera and the computer. Labview with NI Vision Acquisition Software (National Instruments, Austin, TX) was utilized to grab, display, and record the video stream. The NI-IMAQdx High Performance driver interface with an Intel Pro 1000 series network adaptor reduced the CPU processing of network packet data. Using a custom Labview routine, video streams were recorded as uncompressed AVI files to the hard drive. Off-line analysis was used in order to avoid simultaneous image acquisition and real-time analysis that caused frame dropping.

### Particle Tracking and Transcription Rate Analysis

Particles were located in each frame of the movie file using an in-house developed Matlab routine that employs an efficient algorithm based on radial symmetry [19]. The time series of coordinates for each individual bead were stored in a Matlab variable file (MAT) format. The average position of at least two beads stuck on the surface was subtracted from the series of coordinates for each tethered bead to eliminate motion due to mechanical instability. The steps of the data acquisition are shown in S4 Fig. The ensemble of positions for each tethered bead was tested for circular symmetry using the covariance of the Cartesian coordinates. Tethers that did not exhibit symmetry were discarded. For more details refer to Kumar *et*. *al*. [16].

To quantify the elongation rates of individual polymerases, the TPM excursion value of the bead was calculated using the following formula: ${\langle \rho \rangle }_{20s}={\langle \left(\sqrt{{\left(x-{\langle x\rangle }_{20s}\right)}^{2}+{\left(y-{\langle y\rangle }_{20s}\right)}^{2}}\right)\rangle }_{20s}$ where ${\langle x\rangle }_{20s}$ and ${\langle y\rangle }_{20s}$ are the Cartesian coordinates of the attachment point of the bead resulting from a moving average of 20 s, while *x* and *y* are the coordinates of the bead position at different observation times obtained from the particle tracking routine [16]. The time-dependent excursion values were related to the DNA tether length via a previously obtained calibration curve (Figure 4 in [16]). Then, the average rate of each elongation run was obtained by dividing the difference between the final and the initial tether length in the entire elongation trace by the elapsed time. The distribution of the values obtained for all the elongation runs observed was then fitted by a single exponential to determine the characteristic average velocity (R-value = 0.95). Rates between two consecutive data points in each of the 65 elongation runs (S1 File) were also calculated, and smoothed with a ten point moving average. The maximum value of these “instantaneous” rates for each elongation run was noted and a histogram built. The distribution was then fitted with a single exponential to find the characteristic maximum velocity for the 65 tethers (R-value = 0.98).

### Multiplexed DIC

**Fig 2:**
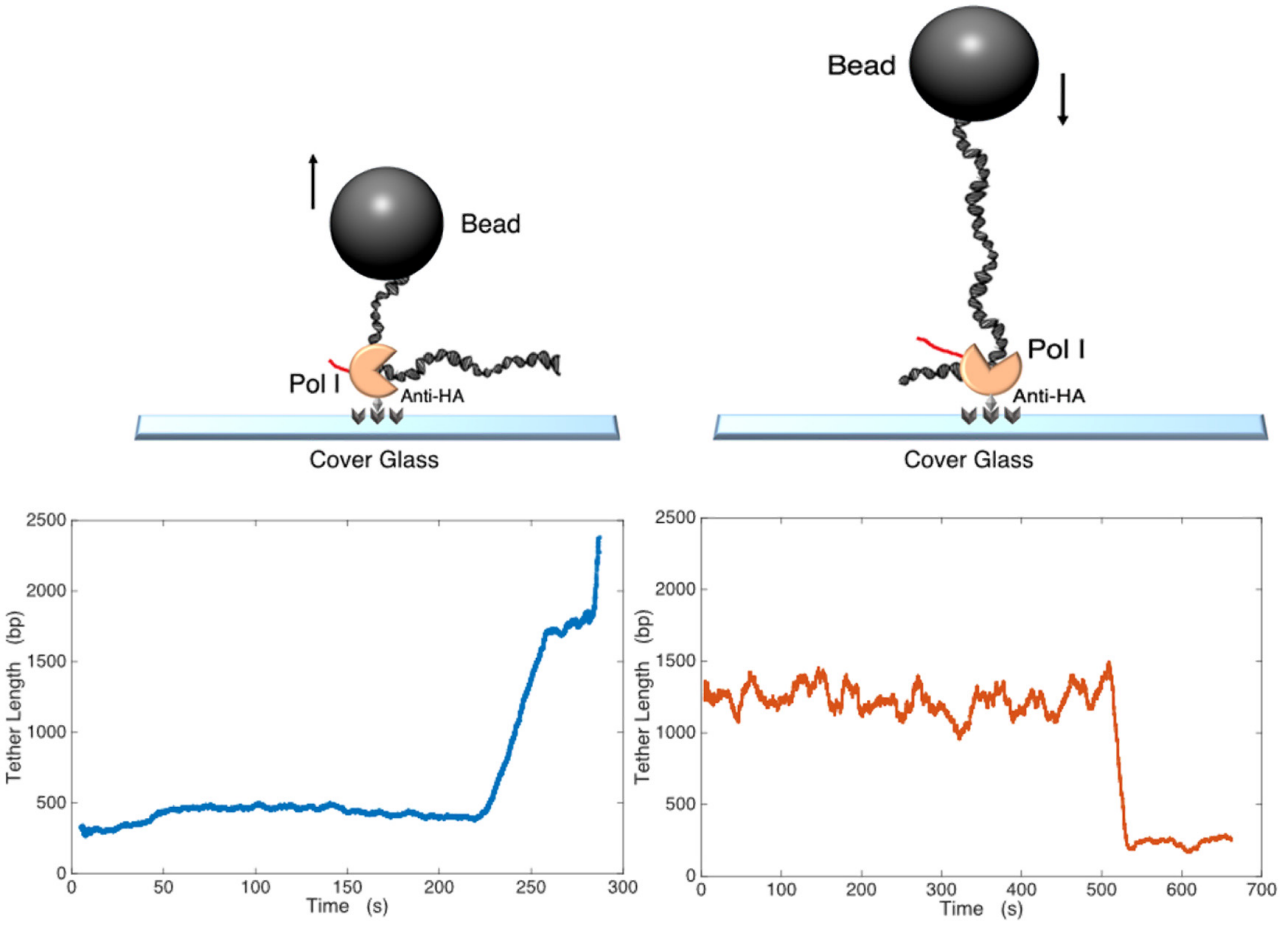
Schematic of experimental configurations and representative elongation traces. (Upper) A 480 nm, anti-dig labelled bead was attached to the upstream (left) or downstream (right) end of the DNA template. The triply hemagglutinin- (3-HA) labelled Pol I of a stalled complex attached to the anti-HA-coated cover glass. (Lower) Tracking of a bead with nm accuracy allowed determination of the amplitude of Brownian motion which is a function of the tether length. After addition of NTPs, elongation resumed, and the DNA tether length increased (left) or decreased (right). Data during intervals in which beads stuck transiently to the surface were discarded.

To compensate for the fact that only approximately 1–2% of the tethered, halted Pol I complexes resumed transcription upon addition of all four NTPs, a high throughput TPM setup was developed by reducing the customary 63X magnification in order to observe up to 100 transcription complexes simultaneously. This yielded 1–2 elongating complexes in average for each experiment (microchamber). Our DIC microscopy measurements were done at a total magnification of 40X, for which the image of a bead with a 240 nm radius was about 8 pixels across (S3 Fig). In the experimental setup of Fig 2 (left), the distribution of the RMS excursion values for DNA tethers of halted complexes (556 bp-long) measured with DIC microscopy is shown in S5 Fig and has a mean of 168.1 ± 25 nm, in accord with previously obtained values. [16]

## Results

### RNA Polymerase I activity can be measured using TPM

Single molecule approaches have yielded several key insights into transcription by *E*. *coli* RNA polymerase and yeast RNA polymerase II [20, 21]. In this study, we present a single-molecule analysis of transcription elongation by eukaryotic RNA polymerase I (Pol I). We used TPM to monitor transcription elongation by single Pol I enzymes on two different DNA templates. In one experimental configuration, we used an rDNA template amplified from yeast that contained 500 bp upstream and 2 kbp downstream of the promoter (S1 Fig, top). The upstream end of DNA was labelled with digoxigenin (dig), for attachement to an anti—dig-labelled bead. Pol I carried a triple hemagglutinin (3-HA) tag on the carboxy terminus of the second largest subunit (A135). This tag served for immobilization of the polymerase on the chamber surface (Fig 2). Using a modified DNA template in which cytosine was replaced with guanine between positions +1 and +56, we initiated transcription by adding 200 μM ATP, GTP and UTP. Elongation complexes stalled at the first position encoding C, +56, as described previously [7], yielding microspheres tethered by 556 bp-long DNA constructs. These beads were tracked, and control data were acquired for 5 minutes to check the initial tether length. Then 200 μM of all four NTPs were added. This substrate concentration has been the standard condition in several previous *in vitro* analyses using Pol I and is thought to be sufficient for maximal transcription elongation rates [22]. During nucleotide addition the image was typically blurry due to liquid flow, but after about 1 minute the image cleared and accurate particle tracking was again possible. From the moment of nucleotide addition, after variable delays, transcription elongation resumed and the DNA tether length increased as shown in Fig 2 (bottom left panel). Additional elongation events are shown in S6 Fig.

A different experimental approach employed a 2 kbp DNA template, labelled with a bead on the downstream end (S1 Fig, bottom, and Fig 2, right). In this configuration, the tether length decreased as transcription elongation progressed (Fig 2, bottom right). Together, these data show that transcription elongation by Pol I on single rDNA segments can be monitored *in vitro* using TPM. Although both experimental designs were effective, we chose the extending configuration shown in the left panel of Fig 2 for all subsequent analyses.

### RNA polymerase I is not highly processive *in vitro*

**Fig 3:**
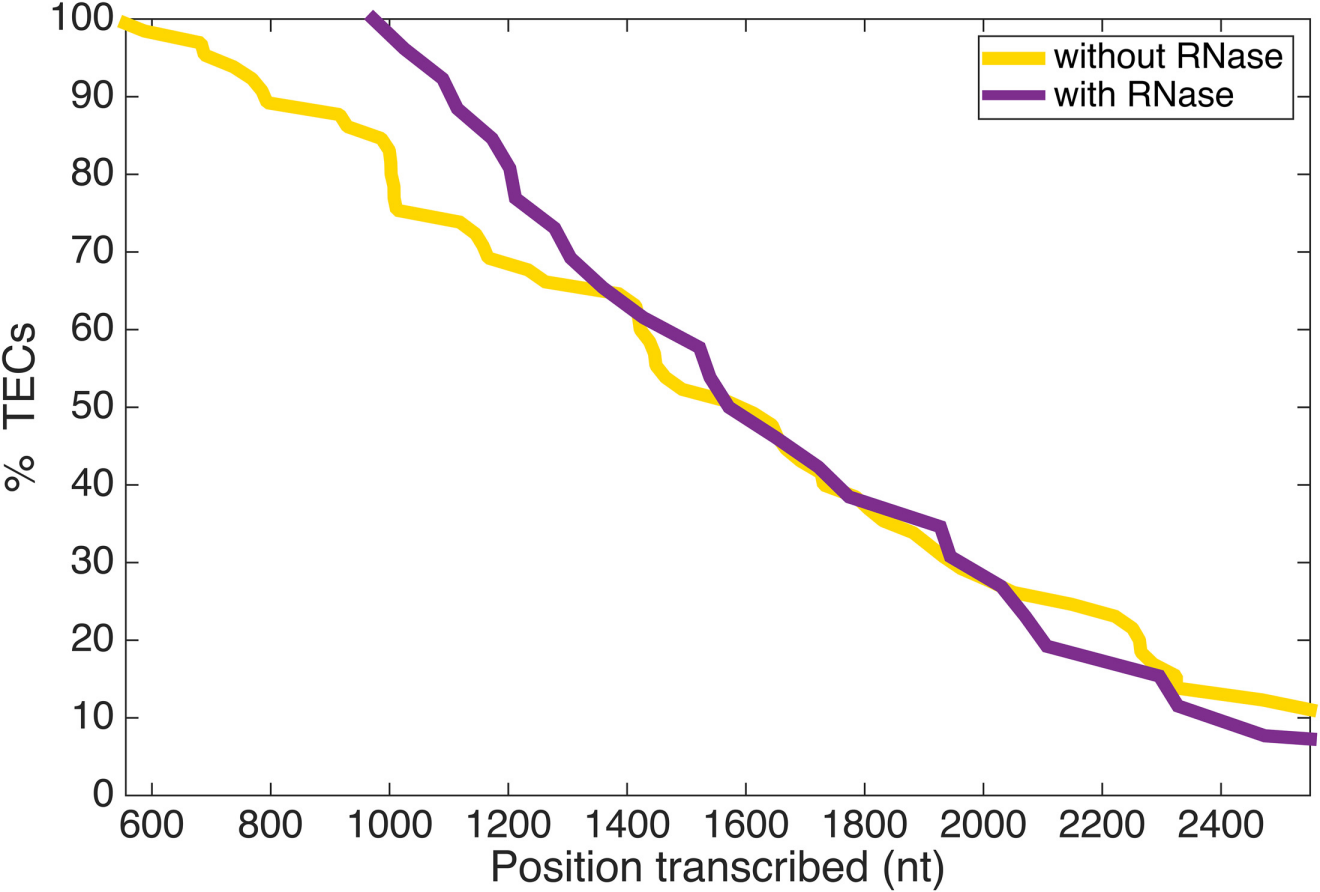
Pol I processivity measured by TPM. Elongation complexes arrested after transcribing different distances along the DNA template, with only seven out of sixty-five complexes reaching the expected run-off site and releasing DNA. The percentage of elongation complexes that reached a given position along the template is plotted for single molecule experiments with (purple) or without (gold) added RNAse. Note that position 556 corresponds to the halt site and 2444 nucleotides can be transcribed before runoff at position 2500.

One somewhat surprising observation evident in the TPM data was that the majority of polymerases did not reach the end of the 2 kbp template. In fact, only 7 out of 65 elongation complexes reached the end (associated with release of the template and bead). Fig 3 (gold curve) is a histogram of the percentage of transcription elongation complexes versus positions transcribed along the template before arresting. In order to distinguish between paused complexes that might resume elongation and terminally arrested complexes, data were recorded for at least 15–25 minutes, approximately ten-fold longer than the time expected to be required for transcription of a 2 kbp template, based on published *in vitro* Pol I transcription data [8]. Defining processivity of a polymerase as the ability of the enzyme to reach the end of the template, our TPM data demonstrate that Pol I is not highly processive *in vitro*. This finding contrasts with observations *in vivo* [23].

**Fig 4:**
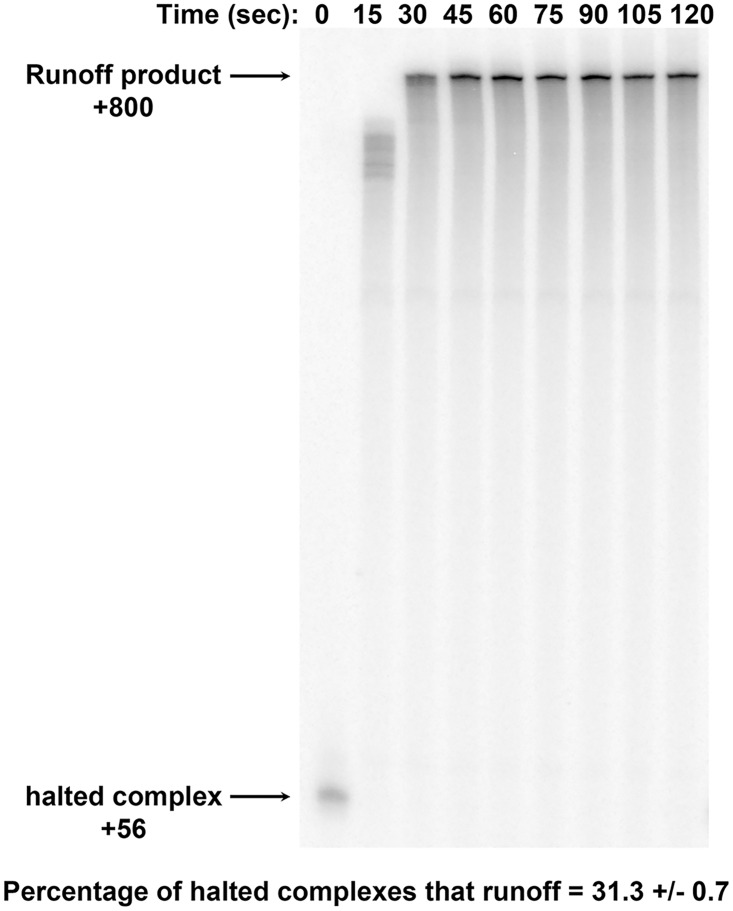
Pol I processivity measured in bulk. Transcription was initiated from a template similar to that described for the TPM measurements described above (Figs 1–3) except there were no dig-labels and the “run off” end point was +800 with respect to the transcription start site. Transcription was initiated in the presence of core factor, TBP, Rrn3 and Pol I, using 200 μM ATP, UTP and 20 μM GTP plus α32P-GTP (10 μCi per 20 μl reaction). Transcription elongation complexes were halted at +56, a sample was collected in phenol, and CTP was added to the remainder to achieve a 200 μM final concentration. Samples were collected into phenol as a function of time, precipitated, resuspended in formamide loading dye and analyzed by denaturing gel electrophoresis and phosphorimaging. This experiment was repeated three times (S3 File) and after accounting for the level of GTP incorporation in the 800 versus the 56 bp transcripts, the average percentage of complexes that reached the end of the template was calculated and is indicated with the standard deviation.

To determine whether this defect in processivity was an artefact of our single-molecule analysis or a general feature of Pol I transcription *in vitro*, we performed bulk biochemical analysis of Pol I transcription with α-32P GTP included in the reactions to monitor transcription as described previously [8]. In this experiment, we clearly observed halted complexes at +56 with respect to the transcription start site and the 800-base runoff product (Fig 4). To measure the fraction of Pol I complexes that reached the end of the template, the band intensities at +56 and +800 were normalized by the number of G residues in the respective products (S3 File). For this calculation, the +800 band was quantified after maximal product accumulation was reached (in the representative gel shown, corresponding bands between 45 seconds and 120 seconds were averaged; Fig 4). The normalized values for +800 were then divided by the values for +56 to estimate the fraction of halted complexes that resumed to produce full-length transcripts (S3 File). When we averaged this value for three independent set of transcription reactions, we found that only 31% of halted complexes resumed to produce full length product. This value is lower but reasonably close to the degree of processivity observed in single molecule assays where 60% of complexes transcribed greater than or equal to 800 base pairs (Fig 3). Together these data demonstrate that under these *in vitro* conditions, Pol I is not as processive as *in vivo*. Thus, it is likely that additional factors enhance the processivity of Pol I *in vivo*, and these factors or conditions remain to be discovered.

### Measurement of Average and Maximum Elongation Rate

**Fig 5:**
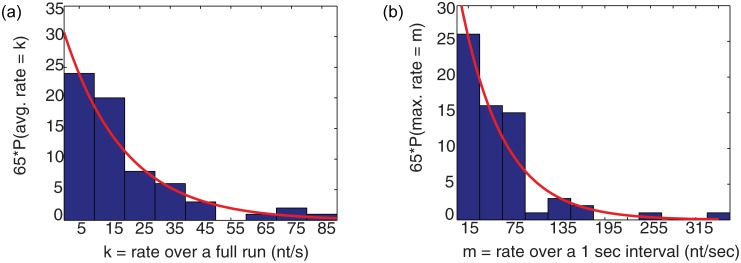
Pol I elongation rates. The average rate (a) was calculated from the beginning to the end of each elongation run. The maximum rate (b) in each of the 65 traces was found as described in the Materials and Methods, “Particle Tracking and Transcription Rate Analysis.” The distributions of (a) and (b) were then fitted with exponential functions with mean values of 20.7 (average) and 58.4 (maximum) nt/s rates of elongation (R-values of 0.95 and 0.98 respectively). These mean values are significantly different with respective 95% confidence intervals of (14.1–27.3) and (48.6–68.4) relative to the fitted mean values. Note that for an exponential distribution, the standard deviation is equal to the mean.

Fig 5a shows the distribution of average elongation rates for each of the 65 events (S1 File) observed in the presence of 200 μM NTPs. Fitting this as an exponential distribution produced a mean value of 20.7 nt/s (95% confidence interval 14.1–27.3). This exactly matches that measured using *in vitro* bulk assays [15]. Similarly fitting the characteristic maximum elongation rates observed in the 65 traces (Fig 5b) as an exponential distribution produced a mean elongation rate of 58.4 nt/s (95% confidence interval 48.6–68.4), which is close to the rate of 60 nt/s estimated for the *in vivo* situation based on electron microscopy studies [14]. Note that high rates in the tail of panel (b) are well within the range observed in vivo [24]. When the concentration of NTPs was decreased to 50 μM, the average elongation rate decreased to 10 nt/s (S1 Table) (with 5 μM the average rate was approx. 4 nt/s, data not shown), as expected based on previous studies using sub-saturating NTP concentrations [15]. Control measurements in the absence of NTPs yielded no net change in tether length over time (zero elongation rate) (S1 Table).

The standard deviation for the TPM signal (RMS excursion) for a 522 bp DNA fragment tethering a 490 nm diameter bead is about ± 8 nm [25]. Using the calibration established by Kumar et al. [16], an 8 nm change in the TPM signal would correspond to a 70 bp translocation by Pol I. At the average rate of transcription such a translocation would require 3.5 seconds and even less than 2 s for the maximal transcription rate. Therefore, elongation events of 70 or more base pairs leading to stable tether length increases might be distinct in our analysis, but elongations by a few tens of bp are not, especially without sufficient time to adequately average the excursion signal [26]. This prohibited an analysis of pauses on the appropriate scale (base pairs). Indeed, pausing was not addressed in previous TPM-based studies of transcription elongation [27]. In the future, transient events might be analyzed by reducing the bead size to increase diffusion and lower the necessary averaging time, or by applying tension with optical or magnetic tweezers if the activity of the tethered complexes can be improved.

### Nascent RNA interferes with Pol I transcription elongation

The greater than two-fold difference between the average elongation rate of Pol I (20 nt/s) and its maximum rate (58 nt/s), suggested that we would have been able to detect sequence-dependent variation. When we re-analyzed the data to find the “local” elongation rates, mean elongation rates for intervals of 100 bp, as a function of position along the template, the average rates determined across these intervals ranged from 10–30 nt/s (S7 Fig, lower). Thus, fast elongation was due to stochastic behaviour and not clearly related to specific sequences.

Note that the fragment used in our TPM assay contains part of the 18S and the 5’ETS sequence of *S*. *cerevisiae* rDNA (Fig 1, lower) and rDNA generally has high GC content compared to the rest of the genome. We conjectured that transient negative supercoiling generated by elongating Pol I in the upstream DNA template, together with the lack of trailing Pol I’s as would be present *in vivo*, might have induced R-loops and interfered with processivity. Therefore, we expected to find lower elongation processivity in GC-rich regions of the sequence, which averages 43% GC content (S7 Fig, upper).

To test this, we measured elongation in the presence of RNase A/T1 (n = 13) which digests and removes any RNA from DNA and protein preparations or RNase H (n = 14) which cleaves the 3’-O-P bond of RNA and eliminates ribonucleotides from DNA/RNA duplexes (S2 File). These experiments suggest that nascent RNA mildly attenuates the maximum elongation rate of Pol I and improves processivity from position 556 to position 1400 of the DNA construct. Neither RNase A/T1 nor RNase H substantially increased the average elongation rates, which were 21 or 19 nt/s respectively (S1 Table), but the maximum rate was slightly lower, 41 nt/s (S8 Fig). However, while about 80% of TECs transcribed through position 1000 of the template without RNase, 100% of TECs elongated at least this far in the presence of RNase (Fig 3).

## Discussion

### Pol I processivity

In yeast, Pol I transcribes one of the longest genes encoded in the genome (~6.7 kb), and previous studies, including visualization of Pol I transcription by electron microscopy, demonstrate that Pol I is highly processive [14, 23]. However, both the TPM data (Fig 3) and bulk transcription data (Fig 4) demonstrate that Pol I has poor processivity (defined as the ability to complete elongation of the entire template) *in vitro* on a naked DNA template. Theoretically, the frequent arrests in TPM measurements might be favoured by slight (femtonewton) tension from the tethered particle [28] opposing transcription (Fig 2B). Though possible, this interpretation is unlikely for two reasons. First, we also implemented the configuration shown in Fig 2A in which tension from the tethered particle would have favoured transcription. Neither experimental setup resulted in efficient elongation to the end of the DNA. Furthermore, the bulk assays shown in Fig 4 employ linear, DNA templates without attached beads. Since Pol I was not fully processive in those assays either, premature transcriptional arrest is not due to the tethered particle.

Poor processivity *in vitro* may indicate that one or several factors that contribute to the exceptional processivity of Pol I *in vivo* are absent in the purified system. A small number of transcription elongation factors that influence Pol I transcription have been described [23, 29–32]. Perhaps one of these factors directly influences the enzyme, just as RNase improved processivity between positions 556 and approximately 1400 of the DNA construct. Our direct assay is ideal to evaluate additional components of the Pol I machinery. Since transcription of ribosomal DNA is gaining interest as a potential chemotherapeutic target [11, 13], characterization of factors that directly affect Pol I activity will have obvious clinical significance.

### Pol I elongation rate

It is interesting that in our simplified experimental conditions, without significant tension on the DNA template or nucleosomes, the characteristic maximum elongation rate of Pol I was similar to that estimated *in vivo* from EM studies [14]. One interpretation of this observation is that actively transcribed rDNA repeats are barrier-free *in vivo*. Alternatively, multiple barriers to Pol I transcription elongation may be encountered *in vivo*, but additional transcription factors aid the polymerase in negotiating these barriers. Previous studies suggest that several transcription elongation factors influence Pol I activity *in vivo* but whether these proteins influence the average velocity of the enzyme remains unclear [29–32].

However, it is interesting to note that the difference we found between characteristic values of the average and maximum elongation rates by RNA Pol I is in perfect agreement with the difference found between previous estimates of the average elongation rate *in vitro* and *in vivo*. *In vivo*, *S*. *cerevisiae* and mammalian Pol I [14, 33] had been reported to elongate at an average speed of 60 nt/s and 91 nt/s, respectively. The value estimated for *S*. *Cerevisiae* is identical to the one we directly measured in our assay for the characteristic maximum elongation rate, and even the value estimated in mammalian cells is consistent with our result as well as the idea that, *in vivo*, the elongation rate is higher than the average rate measured *in vitro*. In this experimental setup, both our measurement and the estimate by Viktorovskaya et al. [8] yield an *in vitro* average elongation rate of 18–20 nt/s. A recent *in vitro* study using optical tweezers by Lisica *et al*. [34] reported, instead, a similar average and maximum elongation rate (~32 nt/s and ~39 nt/s, respectively). Their experimental conditions differed from those used in our work in that they used a DNA template with homogeneous distribution of A-T and G-C content, instead of a segment of the *S*. *cerevisiae* rDNA gene, and a different buffer (20 mM HEPES (pH 7.6 at 20°C), 60 mM (NH_4_)_2_SO_4_, 8 mM Mg_2_SO_4_, 10 μM ZnCl_2_, 10% (wt/vol) glycerol). However, the most likely reason for the lack of difference between the average and maximum elongation rates they observed may be the non-negligible tension applied to the DNA template in their measurements (2–10 pN), while our measurements were essentially without tension.

Our studies indicate that nascent RNA may also have a significant effect on Pol I activity, since enzymes that digest RNA (RNase A/T1) or RNA hybridized to DNA (RNase H) improved processivity (see above) but did not change overall rates of elongation. Such complex effects by nascent RNA might be modulated *in vivo* by simultaneous processing of the nascent rRNA, high Pol I density per gene, and accessory factors; all of which inhibit R-loop formation and favour transcriptional elongation.

Our measurements showed variations in the elongation rates of all the Pol I complexes analysed. In this respect, Pol I behaves similarly to other RNA polymerases which are known to have non-uniform elongation rates [35], with the first direct, single-molecule observation of such heterogeneity in bacterial RNAP by Yin et al [36].

This study represents an early step in the effort to develop and apply new methods to characterize the kinetics of transcription elongation by RNA polymerase I. Although there is growing interest in transcription of rDNA, relatively little is known about the molecular mechanisms of Pol I transcription. Given the extremely low yield with which stalled Pol I complexes restart transcription after addition of NTPs in the TPM chamber, multiplexed TPM, which permits negligible template tension and high throughput, proved advantageous to conduct this investigation on Pol I elongation rate. Using multiplexed magnetic tweezers, which enable a higher spatial resolution, might enable rapid progress in defining novel features of transcription by Pol I, the most robust transcription machinery in a growing eukaryotic cell.

## Supporting Information

S1 Fig DNA Constructs. Diagrams of the DNA constructs used in Fig 2 left (right). Using the digoxigenin label to fix the upstream (downstream) ends of the template DNA, produced an increase (decrease) in tether length.
(DOCX)

S2 Fig TPM Microchamber. An image of a TPM microchamber filled with a solution containing green dye.
(DOCX)

S3 Fig TPM field of view. A sample differential interference contrast field of view at 40 X magnification. *(Inset*: an enlarged view of a selected tethered bead.) The scale bar represents 10 μm.
(DOCX)

S4 Fig Flowchart of the data processing strategy. First, a video stream was acquired at 30 frames/second using a GigE camera. Then, with a custom Labview routine, the video stream was recorded to a hard disk as an uncompressed AVI file. During playback of the movie file, selection of tethered beads and particle tracking was carried out with a Matlab routine to generate time series of the *x*,*y* coordinates for each selected bead. Artifactual mechanical drift was corrected by subtracting the average position of at least two stuck beads within the same field of view at each time point in the series.
(DOCX)

S5 Fig Distribution of TPM data. The distribution of excursion values for 556 bp-long DNA tethers monitored with the 40X, DIC-based TPM microscope.
(DOCX)

S6 Fig Elongation traces. Representative elongation events exhibit the increase in DNA tether length observed during transcription by Pol I.
(DOCX)

S7 Fig AT content and Pol I rate. The percentage of AT in the template mildly correlates with local average rates of elongation. The average GC percentage over 30 bp plotted as a function of template position varies between 40–70 percent (upper). The average rates of elongation measured as a function of position for 100 bp intervals along the template generally rise for 1500 nucleotides and then fall to initial values near the end.
(DOCX)

S8 Fig Maximum rates with RNase. Distribution of the maximum elongation rates in the presence of RNase A/T1 or RNase H.
(DOCX)

S1 File RNase-free Data. Tether length (bp) versus time (s) data for the 65 elongation runs recorded in the absence of RNase and filtered with a moving average spanning 20 s. Time is in column 1 and tether length is in column 2.
(MAT)

S2 File RNase Data. Tether length (bp) versus time (s) data for the 27 elongation runs recorded in the presence of RNase. The data have been averaged over 20 s. Time is in column 1, rho square (nm^2^) in column 2 and tether length in column 3.
(MAT)

S3 File Gel data. RNA Polymerase processivity as measured by gel electrophoresis.
(XLSX)

S1 Table Average Rates. Average elongation rates with 0, 50, or 200 μM NTPs and with or without RNAses.
(DOCX)
