# Supplementary material for: Direct Characterization of Transcription Elongation by RNA Polymerase I
Source: PLoS One. 2016 Jul 25;11(7):e0159527. doi: 10.1371/journal.pone.0159527 (PMC4959687; doi:10.1371/journal.pone.0159527)
Supplement: S1 Fig — Diagrams of the DNA constructs used in Fig 2 left (right). Using the digoxigenin label to fix the upstream (downstream) ends of the template DNA, produced an increase (decrease) in tether length. (DOCX) [file pone.0159527.s001.docx]

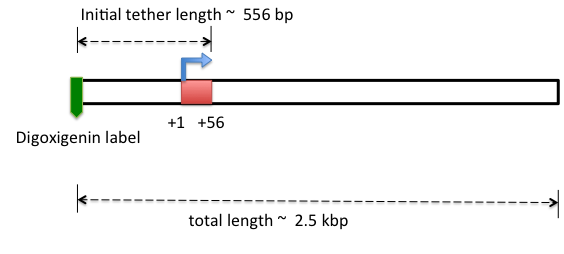


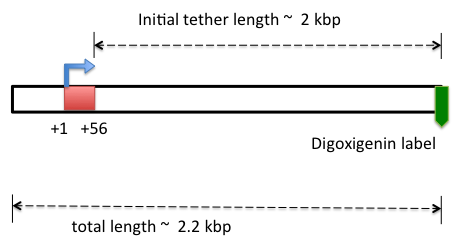


**S1 Fig. DNA Constructs.** Diagrams of the DNA templates used in Figure 2A (upper) and 2B (lower). Using the digoxigenin label to fix the upstream (downstream) ends of the template DNA, produced an increase (decrease) in tether length.
