# Supplementary material for: Direct Characterization of Transcription Elongation by RNA Polymerase I
Source: PLoS One. 2016 Jul 25;11(7):e0159527. doi: 10.1371/journal.pone.0159527 (PMC4959687; doi:10.1371/journal.pone.0159527)
Supplement: S2 Fig — An image of a TPM microchamber filled with a solution containing green dye. (DOCX) [file pone.0159527.s002.docx]

22 X 22 mm coverslip above


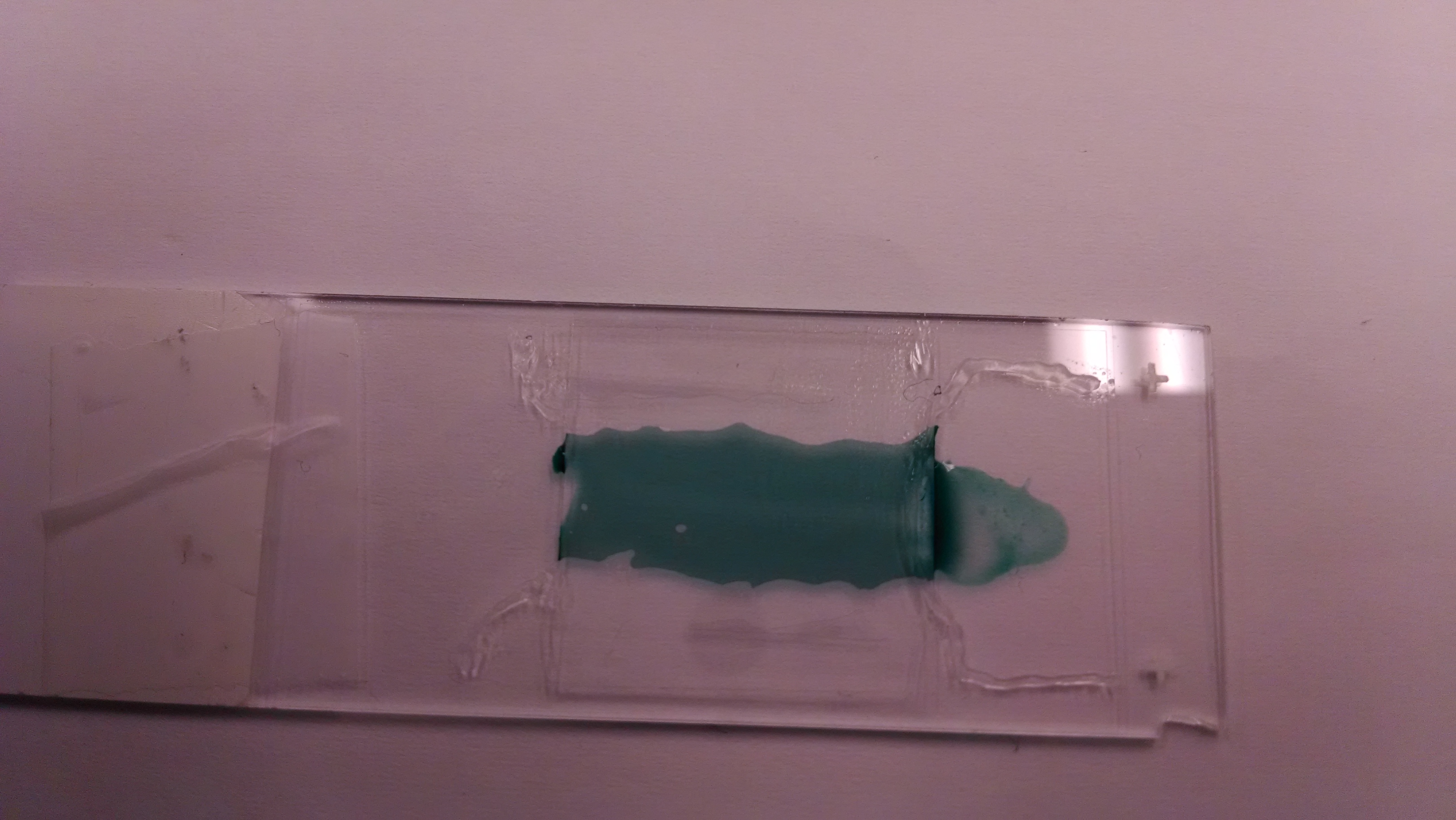


50 X 24 mm coverslip below

Double-sided tape

Vacuum grease between coverslips

20-25 μl microchamber

**S2 Fig. TPM Microchamber.** An image of a TPM microchamber filled with a solution containing green dye.
