# Supplementary material for: Direct Characterization of Transcription Elongation by RNA Polymerase I
Source: PLoS One. 2016 Jul 25;11(7):e0159527. doi: 10.1371/journal.pone.0159527 (PMC4959687; doi:10.1371/journal.pone.0159527)
Supplement: S3 Fig — A sample differential interference contrast field of view at 40 X magnification. (Inset: an enlarged view of a selected tethered bead.) The scale bar represents 10 μm. (DOCX) [file pone.0159527.s003.docx]

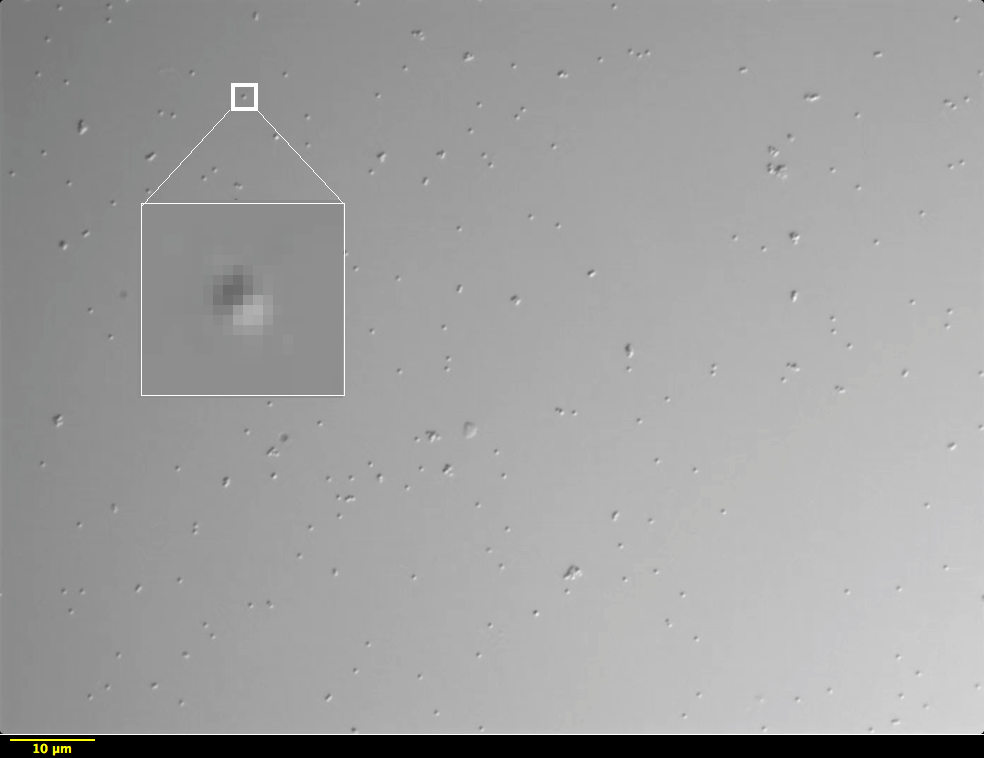


**S3 Fig. TPM field of view**. A sample differential interference contrast field of view at 40 X magnification. *(Inset*: an enlarged view of a selected tethered bead.) The scale bar represents 10 μm.
