# Supplementary material for: Direct Characterization of Transcription Elongation by RNA Polymerase I
Source: PLoS One. 2016 Jul 25;11(7):e0159527. doi: 10.1371/journal.pone.0159527 (PMC4959687; doi:10.1371/journal.pone.0159527)
Supplement: S4 Fig — First, a video stream was acquired at 30 frames/second using a GigE camera. Then, with a custom Labview routine, the video stream was recorded to a hard disk as an uncompressed AVI file. During playback of the movie file, selection of tethered beads and particle tracking was carried out with a Matlab routine to generate time series of the x,y coordinates for each selected bead. Artifactual mechanical drift was corrected by subtracting the average position of at least two stuck beads within the same field of view at each time point in the series. (DOCX) [file pone.0159527.s004.docx]

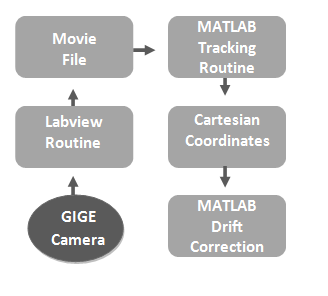


**S4 Fig**. **Flowchart of the data processing strategy.** First, a video stream was acquired at 30 frames/second using a GigE camera. Then, with a custom Labview routine, the video stream was recorded to a hard disk as an uncompressed AVI file. During playback of the movie file, selection of tethered beads and particle tracking was carried out with a Matlab routine to generate time series of the *x,y* coordinates for each selected bead. Artifactual mechanical drift was corrected by subtracting the average position of at least two stuck beads within the same field of view at each time point in the series.
