# Supplementary material for: Direct Characterization of Transcription Elongation by RNA Polymerase I
Source: PLoS One. 2016 Jul 25;11(7):e0159527. doi: 10.1371/journal.pone.0159527 (PMC4959687; doi:10.1371/journal.pone.0159527)
Supplement: S5 Fig — The distribution of excursion values for 556 bp-long DNA tethers monitored with the 40X, DIC-based TPM microscope. (DOCX) [file pone.0159527.s005.docx]

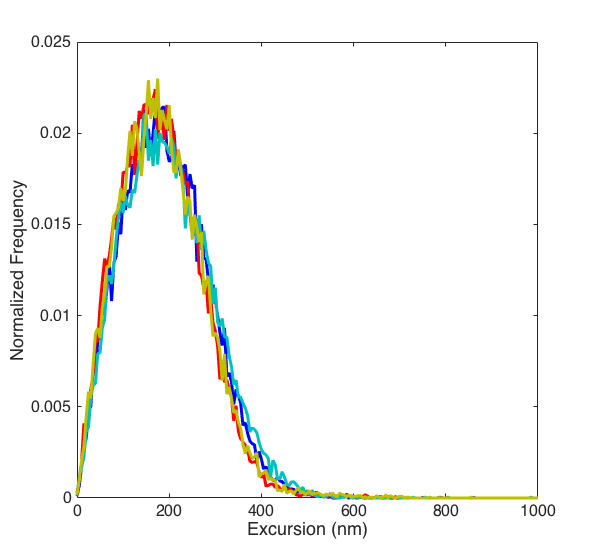


**S5 Fig. Distribution of TPM data.** The distribution of excursion values for 556 bp-long DNA tethers monitored with the 40X, DIC-based TPM microscope.
