# Supplementary material for: Direct Characterization of Transcription Elongation by RNA Polymerase I
Source: PLoS One. 2016 Jul 25;11(7):e0159527. doi: 10.1371/journal.pone.0159527 (PMC4959687; doi:10.1371/journal.pone.0159527)
Supplement: S6 Fig — Representative elongation events exhibit the increase in DNA tether length observed during transcription by Pol I. (DOCX) [file pone.0159527.s006.docx]

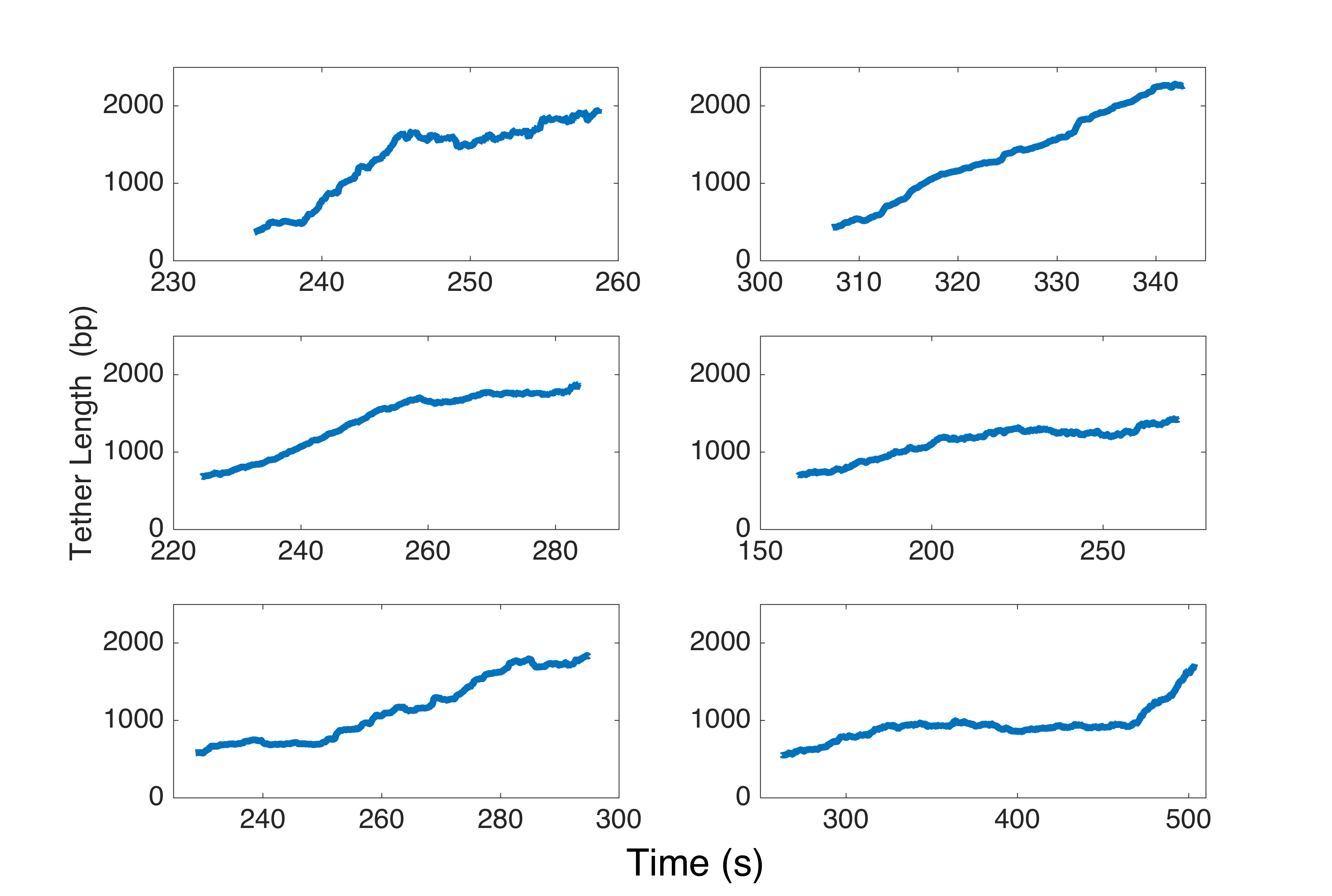


**S6 Fig. Elongation traces.** Representative elongation events exhibit the increase in DNA tether length observed during transcription by Pol I.
