# Supplementary material for: Direct Characterization of Transcription Elongation by RNA Polymerase I
Source: PLoS One. 2016 Jul 25;11(7):e0159527. doi: 10.1371/journal.pone.0159527 (PMC4959687; doi:10.1371/journal.pone.0159527)
Supplement: S7 Fig — The percentage of AT in the template mildly correlates with local average rates of elongation. The average GC percentage over 30 bp plotted as a function of template position varies between 40–70 percent (upper). The average rates of elongation measured as a function of position for 100 bp intervals along the template generally rise for 1500 nucleotides and then fall to initial values near the end. (DOCX) [file pone.0159527.s007.docx]

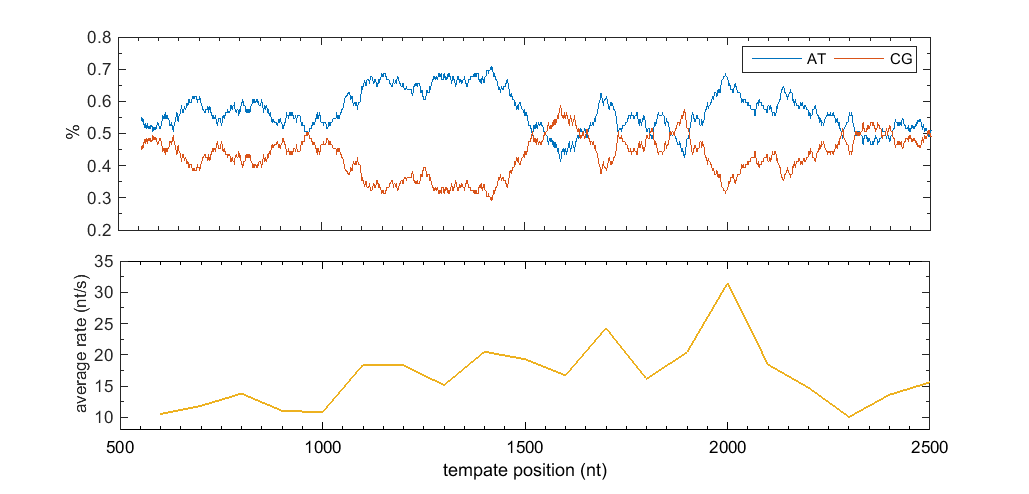


**S7 Fig. AT content and Pol I rate.** The percentage of AT in the template mildly correlates with local average rates of elongation. The average GC percentage over 30 bp plotted as a function of template position varies between 40-70 percent (upper). The average rates of elongation measured as a function of position for 100 bp intervals along the template generally rise for 1500 nucleotides and then fall to initial values near the end.
