# Supplementary material for: Direct Characterization of Transcription Elongation by RNA Polymerase I
Source: PLoS One. 2016 Jul 25;11(7):e0159527. doi: 10.1371/journal.pone.0159527 (PMC4959687; doi:10.1371/journal.pone.0159527)
Supplement: S8 Fig — Distribution of the maximum elongation rates in the presence of RNase A/T1 or RNase H. (DOCX) [file pone.0159527.s008.docx]

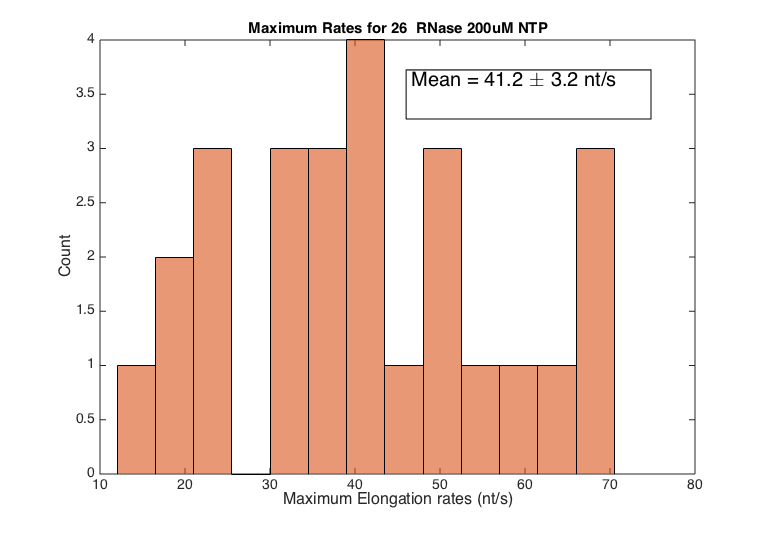


**S8 Fig.** **Maximum rates with RNase.** Distribution of the maximum elongation rates in the presence of RNase A/T1 or RNase H.
