# Supplementary material for: Direct Characterization of Transcription Elongation by RNA Polymerase I
Source: PLoS One. 2016 Jul 25;11(7):e0159527. doi: 10.1371/journal.pone.0159527 (PMC4959687; doi:10.1371/journal.pone.0159527)
Supplement: S1 Table — Average elongation rates with 0, 50, or 200 μM NTPs and with or without RNAses. (DOCX) [file pone.0159527.s012.docx]

|  | **RNAse** | **-** | **-** | **H** | **A/T1** |
| --- | --- | --- | --- | --- | --- |
|  | **[NTP] (uM)** | **0** | **50** | **200** | **200** |
|  |  | **Average elongation rate  (nt/s)** | | | |
|  | **mean** | **0.1** | **10.3** | **18.6** | **21.4** |
| **molecule ID** | **Std. Dev.** | **0.4** | **0.6** | **15.4** | **11.9** |
| **1** |  | **0** | **11** | **8.2** | **18.2** |
| **2** |  | **-0.4** | **10** | **22.3** | **49.3** |
| **3** |  | **0.9** | **10** | **24.6** | **19.0** |
| **4** |  | **0.3** |  | **11.6** | **19.3** |
| **5** |  | **0.1** |  | **7.9** | **37.7** |
| **6** |  | **0.3** |  | **11.0** | **21.3** |
| **7** |  | **-0.2** |  | **17.0** | **9.8** |
| **8** |  | **-0.1** |  | **7.5** | **11.6** |
| **9** |  | **-0.1** |  | **54.0** | **7.9** |
| **10** |  | **-0.1** |  | **3.3** | **15.1** |
| **11** |  |  |  | **21.4** | **15.9** |
| **12** |  |  |  | **48.5** | **33.7** |
| **13** |  |  |  | **4.5** | **19.9** |
| **14** |  |  |  | **19.2** |  |

**Table S1**. **Average Rates**. Average elongation rates with 0, 50, or 200 µM NTPs and with or without RNAses.
